# Philanthotoxin-343 attenuates retinal and optic nerve injury, and protects visual function in rats with N-methyl-D-aspartate-induced excitotoxicity

**DOI:** 10.1371/journal.pone.0236450

**Published:** 2020-07-24

**Authors:** Muhammad Fattah Fazel, Izuddin Fahmy Abu, Mohamad Haiqal Nizar Mohamad, Renu Agarwal, Igor Iezhitsa, Nor Salmah Bakar, Norsham Juliana, Ian R. Mellor, Henrik Franzyk

**Affiliations:** 1 Institute of Medical Science Technology, Universiti Kuala Lumpur, Kuala Lumpur, Malaysia; 2 School of Medicine, International Medical University, Bukit Jalil, Kuala Lumpur, Malaysia; 3 Faculty of Medicine, Universiti Teknologi MARA, Sungai Buloh, Selangor, Malaysia; 4 Faculty of Medicine & Health Sciences, Universiti Sains Islam Malaysia, Indah, Kuala Lumpur, Malaysia; 5 School of Life Sciences, Faculty of Medicine & Health Sciences, University of Nottingham, Nottingham, United Kingdom; 6 Department of Drug Design and Pharmacology, Faculty of Health and Medical Sciences, University of Copenhagen, Copenhagen, Denmark; National Eye Centre, UNITED STATES

## Abstract

Retinal ganglion cell (RGC) loss and optic neuropathy, both hallmarks of glaucoma, have been shown to involve N-methyl-D-aspartate receptor (NMDAR)-mediated excitotoxicity. This study investigated the neuroprotective effects of Philanthotoxin (PhTX)-343 in NMDA-induced retinal injury to alleviate ensuing visual impairments. Sprague-Dawley rats were divided into three; Group I was intravitreally injected with phosphate buffer saline as the control, Group II was injected with NMDA (160 nM) to induce retinal excitotoxic injury, while Group III was injected with PhTX-343 (160 nM) 24 h prior to excitotoxicity induction with NMDA. Rats were subjected to visual behaviour tests seven days post-treatment and subsequently euthanized. Rat retinas and optic nerves were subjected to H&E and toluidine blue staining, respectively. Histological assessments showed that NMDA exposure resulted in significant loss of retinal cell nuclei and thinning of ganglion cell layer (GCL). PhTX-343 pre-treatment prevented NMDA-induced changes where the RGC layer morphology is similar to the control. The numbers of nuclei in the NMDA group were markedly lower compared to the control (p<0.05). PhTX-343 group had significantly higher numbers of nuclei within 100 μm length and 100 μm^2^ area of GCL (2.9- and 1.7-fold, respectively) compared to NMDA group (p<0.05). PhTX-343 group also displayed lesser optic nerve fibres degeneration compared to NMDA group which showed vacuolation in all sections. In the visual behaviour test, the NMDA group recorded higher total distance travelled, and lower total immobile time and episodes compared to the control and PhTX-343 groups (p<0.05). Object recognition tests showed that the rats in PhTX-343 group could recognize objects better, whereas the same objects were identified as novel by NMDA rats despite multiple exposures (p<0.05). Visual performances in the PhTX-343 group were all comparable with the control (p>0.05). These findings suggested that PhTX-343 inhibit retinal cell loss, optic nerve damage, and visual impairments in NMDA-induced rats.

## Introduction

Glaucoma is one of the leading cause of irreversible blindness globally [1–4]. It could be categorized into primary open angle, normal tension, angle closure, and congenital glaucoma [5]. The prevalence of glaucoma varies among people from different ethnicities and regions. Primary open angle glaucoma is more prevalent among people of African and European descent, whereas primary angle closure glaucoma is more common in Asian countries especially China. Normal tension, a subtype of primary open angle glaucoma, affects more people in East Asia compared to Caucasians or individuals of African origin [1, 6]. Interestingly, it has been reported that women are at higher risk of developing normal tension and acute angle closure glaucoma compared to men [2]. In 2013, 64 million people suffered from the disease, and with the aging world population, the number was postulated to increase to 80 million by 2020, and 112 million by 2040 [7, 8].

Primary open angle glaucoma has a multifactorial etiology, and the “cupping” of the optic disc has been described as a distinguished clinical feature of the disease [9]. The progressive optic nerve damage seen in this disease often leads to the patients’ loss of visual field which ultimately lead to blindness [10].

Previous studies have demonstrated that the increased activity of N-methyl-D-aspartate (NMDA) receptors appears to be implicated in the pathogenesis of glaucoma. NMDA receptors are important in the synaptic transmission in the central nervous system where its NR2A subunit has been shown to be abundantly distributed in the ganglion cell layer (GCL) of the retina [11–13]. Overstimulation of NMDA receptors has been associated with increased intracellular calcium ion concentration, which culminates into damage to retinal ganglion cells (RGC) and their axons in the optic nerve [12, 14].

Currently available management therapies for glaucoma are directed towards the reduction of intraocular pressure (IOP) [9]. Although elevated IOP is considered as the most important risk factor in this disease, glaucomatous changes may occur despite normal IOP, and hence many patients continue to develop RGC loss regardless of a reduction in IOP [9, 15, 16]. Thus, new therapies that can protect against RGC apoptosis, while providing a simultaneous IOP reduction, will be valuable for arresting the disease.

Previous studies using NMDA receptor antagonists have not shown significant protective effects in human studies despite showing the benefits in animal model, and the reasons for such contradictory results have been widely reviewed [17]. Accordingly, targeting NMDA receptors is likely to yield beneficial outcomes, and these outcomes may in fact be dependent on the type of drug-NMDA receptors interaction, which possibly allows its closure only to suppress excitotoxicity without interfering with physiological excitatory neurotransmission. In this regard, philanthotoxin (PhTX) is a potential candidate. PhTX, a low-molecular-weight toxin from wasps, has been shown to block both NMDA and nicotinic acetylcholine receptor channels non-competitively [18–20]. PhTX-433 is the natural compound isolated from the venom sac of the female wasp, *Philanthus triangulum*, mainly found in the Sahara Desert. The compound, first discovered by T. Piek and colleagues, contains a butyryl/tyrosyl/polyamine structure and exerts glutamate receptor-blocking properties including antagonism towards the NMDA receptors [21–23]. Subsequently, its analogue, known as PhTX-343 (numerals denote the number of methylenes between the amino group of the spermine moiety from left to right) was synthesized and shown to retain the pharmacological properties [18, 20, 24, 25]. The present study aimed to assess the inhibitory effects of PhTX-343 against NMDA receptor-mediated excitotoxicity resulting in neuroprotective effects in rat retinas.

## Materials and methods

### Animals

The study was carried out in accordance with The Association for Research in Vision and Ophthalmology (ARVO) statement for use of animals for ophthalmic and vision research. However, bilateral injections are made in this study to enable the evaluation of visual functions. The study obtained ethical approval by the Animal Ethics Committee, Institute of Medical Science Technology, Universiti Kuala Lumpur (approval number: AEC/MESTECH-UNIKL/2018/001), and The Committee on Animal Research and Ethics, Universiti Teknologi MARA (approval number: UiTM CARE:239/2/2018(6/4/2018)). Male Sprague-Dawley rats (200–250 g) were housed under standard animal house conditions, and 12 h light-dark cycle with food and water available *ad libitum*. Pine shaving bedding were used and change three times per week. Rats were acclimatized to laboratory conditions for one week and were subjected to general systemic and ophthalmic examination. Those found normal were included in the study while those with any ocular abnormalities were excluded.

### Study design

The rats were randomly divided equally into three groups consisting of 12 rats each; Group I was administered with phosphate buffer saline (PBS) which served as the control group, Group II was injected with NMDA compound (Sigma-Aldrich) to induce excitotoxic retinal injury, and Group III was treated with PhTX-343 (University of Copenhagen, Denmark) followed by NMDA-induced retinal injury 24 h later.

All treatments were given bilaterally and intravitreally. Prior to injection, rats were anaesthetized via intraperitoneal injection of ketamine (80 mg/kg) and xylazine (12 mg/kg) mixture. For the intravitreal injections, NMDA and PhTX-343 were diluted in 0.1 M of PBS to obtain a final concentration of 160 nM. Injections were administered with a 26-gauge 10 μl Hamilton syringe. The total injection volume was 2 μl and administered with the aid of a dissecting microscope. The intravitreal injections were made 1 mm from the superotemporal quadrant of the dorsal limbus area performed slowly over 2 min to avoid any significant pressure-induced retinal damages. Polymyxin and neomycin ointments were applied after injection to avoid inflammation and infection [14, 26]. Post-intravitreal injection, none of rats were found to develop any intraocular complications such as cataract or infection.

**Fig 1:**
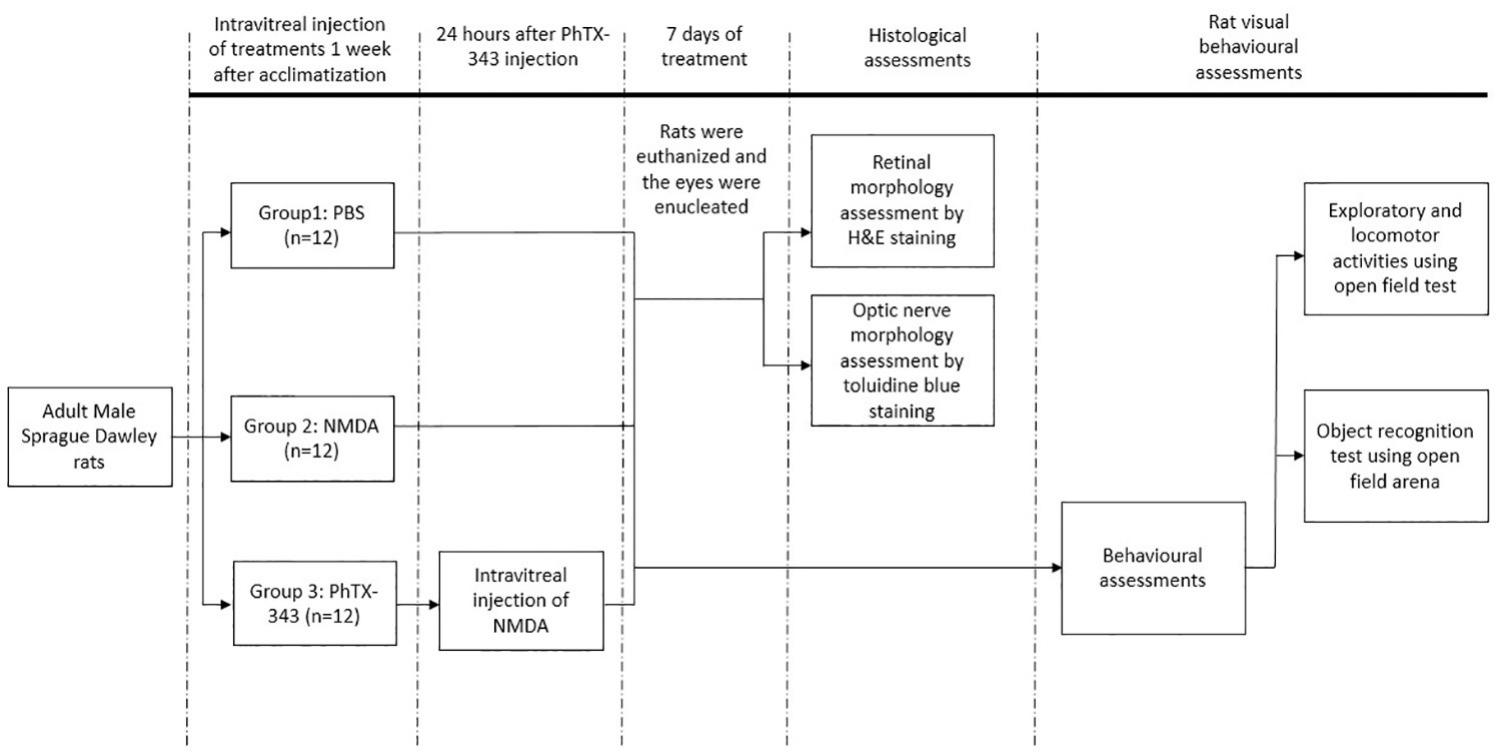
Study design.

Seven days post-injection, the rats were subjected to visual behaviour assessments by using an open field arena. To harvest the retinal and optic nerve tissues, rats were sacrificed by an overdose of ketamine and xylazine mixture (>80 mg/kg and >12 mg/kg, respectively) in accordance to animal ethical guidelines. Retinal tissues were subjected to hematoxylin and eosin (H&E) staining to study the morphological changes by calculating the retinal cell density per 100 μm^2^ or GCL area, and per 100 μm length of GCL. The optic nerves were isolated and subjected to toluidine blue staining to assess the extent of optic nerve damage. The overall study design is depicted in Fig 1.

### Assessment of retinal morphology by using H&E staining

The lenses were removed from the enucleated rat eyes and the retinas were isolated. The retinal tissues were processed through a series of graded alcohol for at least 22 h, and then embedded in paraffin wax. Retinal sections of 3 μm thickness were taken 1 mm from the temporal edge of the optic disc. The sections were deparaffinized, stained with H&E according to standard protocol, and examined at 20× magnification under light microscopy. Three fields of view were randomly chosen, and the images of the sections were saved (in jpeg format). Retinal morphometric assessments were performed with Image J software (National Institutes of Health, Bethesda, MD, USA). The parameters measured included the number of retinal cells in the area (μm^2^) and length (μm) of GCL in the field of view. These measurements were used to calculate the linear and numeric density of retinal cells in GCL [14, 26].

### Assessment of optic nerve morphology by using toluidine blue staining

The optic nerves of rat eyes were cut 1 mm behind the eyeball and subjected to fixation overnight in 10% formaldehyde. Subsequently, the tissues were processed through a series of graded concentrations of alcohol and embedded in paraffin. Sections of 1 mm thickness were cut, deparaffinized and subjected to staining with 1% toluidine blue according to standard protocol. Optic nerve morphology was assessed at 40× magnification under light microscopy. The morphological changes in optic nerve were graded as previously described: Grade 1 = normal optic nerve morphology; Grade 2 = moderate degeneration of axons in a focal area; Grade 3 = widespread degeneration of axons; Grade 4 = all axons showing degeneration [14, 26, 27].

### Assessment of exploratory and locomotor activities of rats in open field test

The visual behaviour of rats after intravitreal injections was determined in an open field arena that allows observation in relation to stress and anxiety-related changes in exploratory and locomotor activities. Rats were expected to exhibit stress and anxiety-related behaviour in open field if their vision were altered. This method was conducted according to a previously validated protocol [28, 29]. The open field arena consisted of a PVC square apparatus (100 cm [L] × 70 cm [H] × 100 cm [W]), and the experiment was conducted in a soundproof room. A camera was installed above the arena for recording of animal activities. Each rat was placed in the centre of the square, and was let free to explore the arena for 10 min (trial 1). This was followed by object recognition training and testing phase with object displacement and replacement (further described below). The total distance travelled, total immobile time and total immobile episodes for each rat were recorded. To prevent transmission of olfactory cues, the arena was swabbed with 75% ethanol solution between trials. All parameters were analyzed and recorded using ANY-maze software (Stoelting Co., USA) [28–31].

### Object recognition test using open field arena

**Fig 2:**
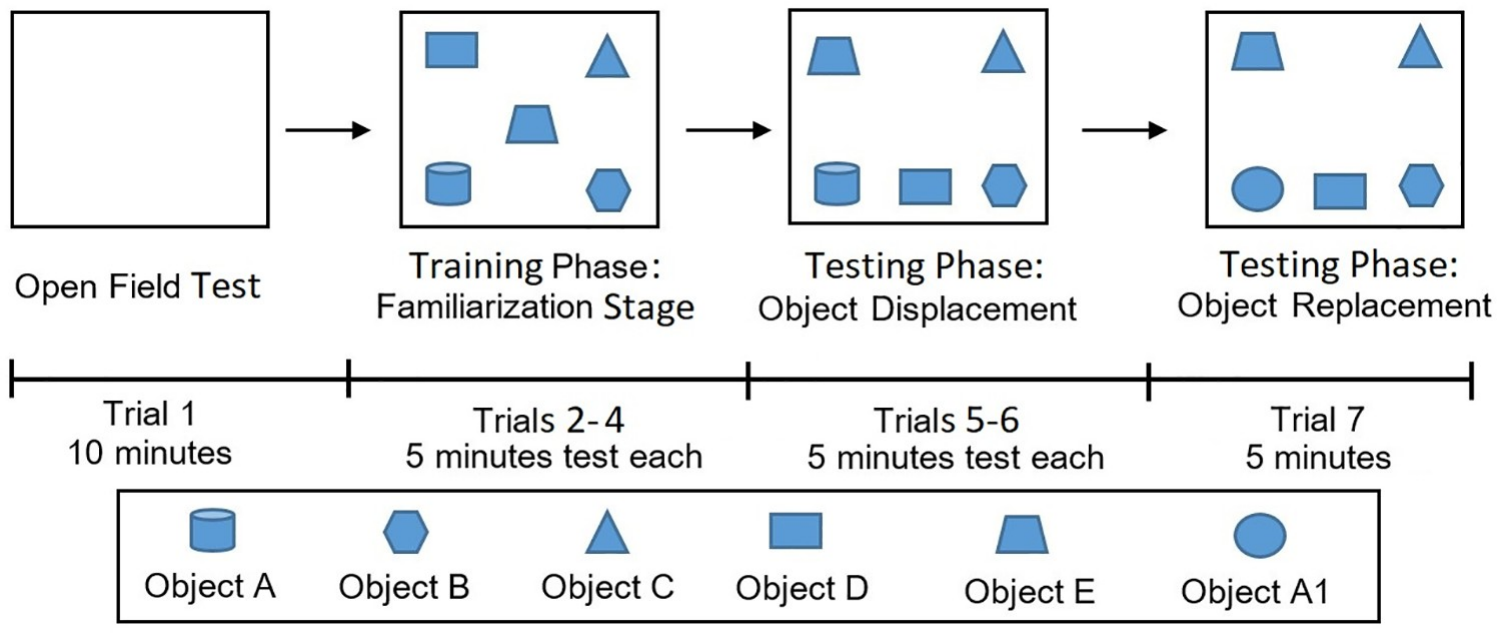
Design of object placements in the open field arena.

The experiment comprised of two parts, training phase (trials 2–4) and testing phase (trials 5–7), which involve object displacement and replacement. Six objects were used in this study: (A) glass cylindrical water bottle with a rounded end (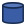), (B) hexagon-shaped bottle (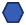), (C) cone-shaped bottle (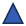), (D) cube-shaped bottle (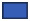), (E) jam jar (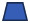), and (A1) sphere jar (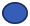). After trial 1 (10 min of habituation time in the open field arena), objects (A-E) were placed in the arena and the rats were then allowed to explore around the five objects (trial 2–4). This phase is also called the familiarization phase. Then, in the subsequent trials 5 and 6 (object displacement test), object D (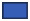) was removed and replaced with object E (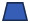), then object D (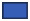) was positioned between objects A (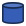) and B (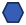). This is followed with trial 7 (object replacement test) where object A (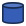) was replaced with a novel object A1 (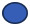). Trials 2–7 were conducted for 5 min each. The movements and feedback from the rats were recorded and analyzed by using the ANY-maze software. The arena was swabbed with 75% ethanol solution before each trial to prevent transmission of olfactory cues [28, 29]. Extra time spent at the novel object indicates their innate preference for novelty. However, it was assumed that intravitreal injection of NMDA had no central effect and the failure to recognize novel objects were mainly due to reduced vision. The experimental procedure and the placement of objects is illustrated in Fig 2.

### Statistical analysis

All data were expressed as mean ± standard error of the mean (SEM). After determination of normality distribution by Shapiro-Wilk test, statistical significance for retinal morphological analysis was assessed by using one-way analysis of variance (ANOVA) followed by post-hoc, Tukey’s test for multiple comparisons performed on GraphPad Prism v8 (GraphPad Software., San Diego, CA). For optic nerve morphology and visual behaviour analysis, the data was non-parametric, hence were statistically analyzed with Kruskal-Wallis test, followed by Dunn’s multiple comparison test using SPSS 22.0 for Windows (SPSS Inc., Chicago, IL.). P values of less than 0.05 were considered statistically significant for all analysis.

## Results

### Effects of PhTX-343 on retinal morphology

**Fig 3:**
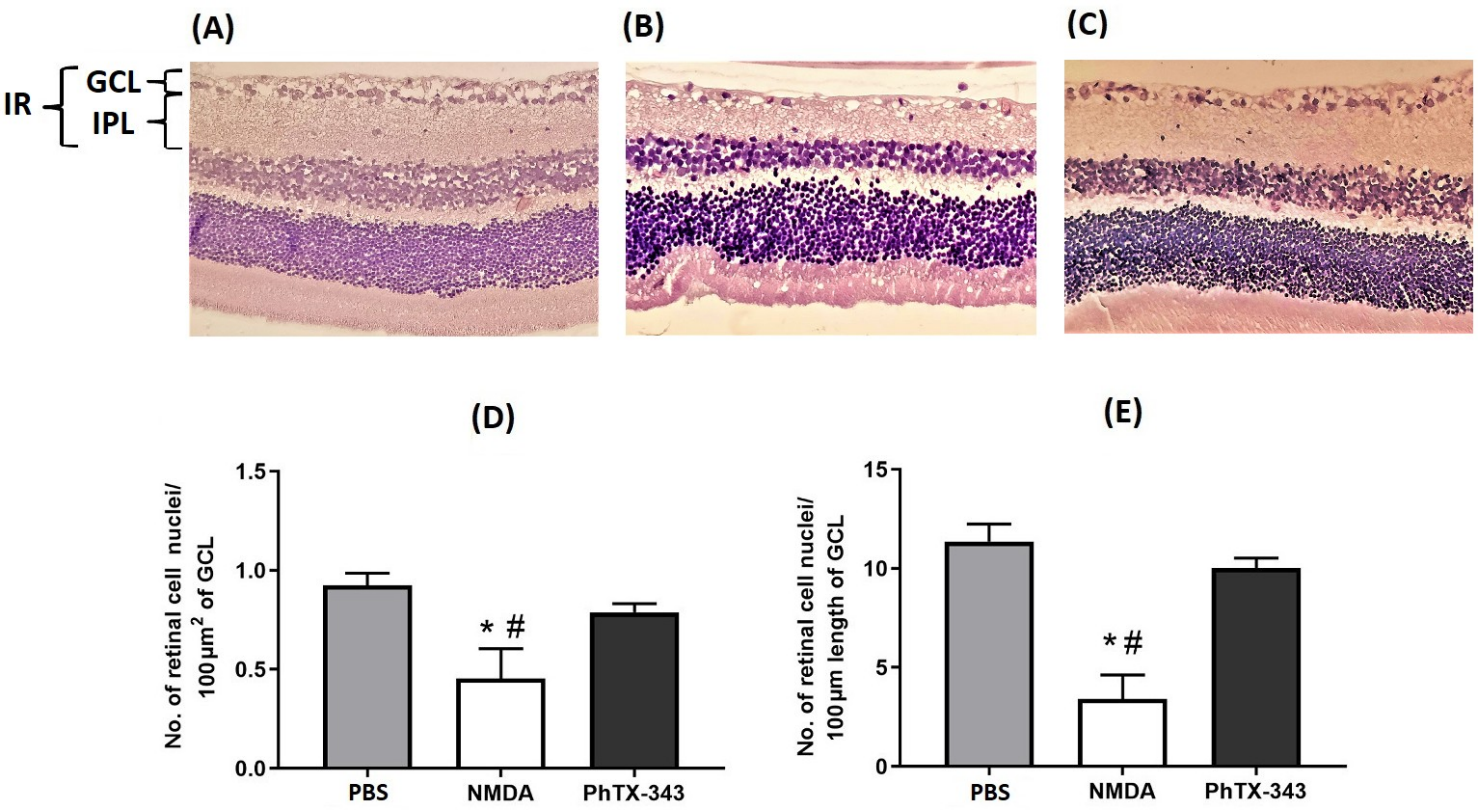
Photomicrographs of retinal sections from each group stained with hematoxylin and eosin (H&E) seven days after intravitreal injections. (A) PBS group presenting intact GCL, (B) NMDA group showing broad loss of retinal cell nuclei, (C) PhTX-343 pre-treatment group followed by NMDA administration after 24 h showing minimal loss of ganglion cells. The histological assessments were quantified with morphometric analysis and presented as a bar graph: (D) number of retinal cell nuclei per 100 μm^2^ of GCL area, (E) number of retinal cell nuclei per 100 μm length of GCL. Data were analyzed using one-way ANOVA followed by post-hoc Tukey’s multiple comparisons test: *p<0.05 versus PBS; #p<0.05 versus PhTX-343 (Scale bar 100 μm). GCL: ganglion cell layer, IPL: inner plexiform layer, IR: inner retina layer.

Retinal sections stained with H&E as displayed in Fig 3 showed a significant loss of retinal cell nuclei and GCL thinning in Group II (NMDA-treated rats). The retinas of rats in the group that received pre-treatment with PhTX-343 (Group III) displayed similar morphology to Group I (control group administered with PBS). Quantitative retinal morphometric analysis (the number of retinal cell nuclei counted within 100 μm^2^ area and along 100 μm length) was performed to further assess the degree of retinal cell loss. The NMDA-treated rats showed a significant low number of retinal cell nuclei/100 μm^2^ area of GCL as compared to that of control rats, corresponding to a 2.0-fold difference (p<0.05). In contrast, the cell density for the PhTX-343 pre-treated rats (Group III) was 1.7-fold higher (p<0.05) than found for the NMDA-treated Group II. The number of retinal cell nuclei/100 μm length in Group I and III were also significantly higher (p<0.05) by 3.3 and 2.9 folds, respectively, as compared to that found for the NMDA-treated Group II. Both parameters showed no significant differences between Group I and III, indicating that PhTX-343 pre-treatment protected the rat retinas from NMDA-induced injury.

### Effects of PhTX-343 on optic nerve morphology

**Fig 4:**
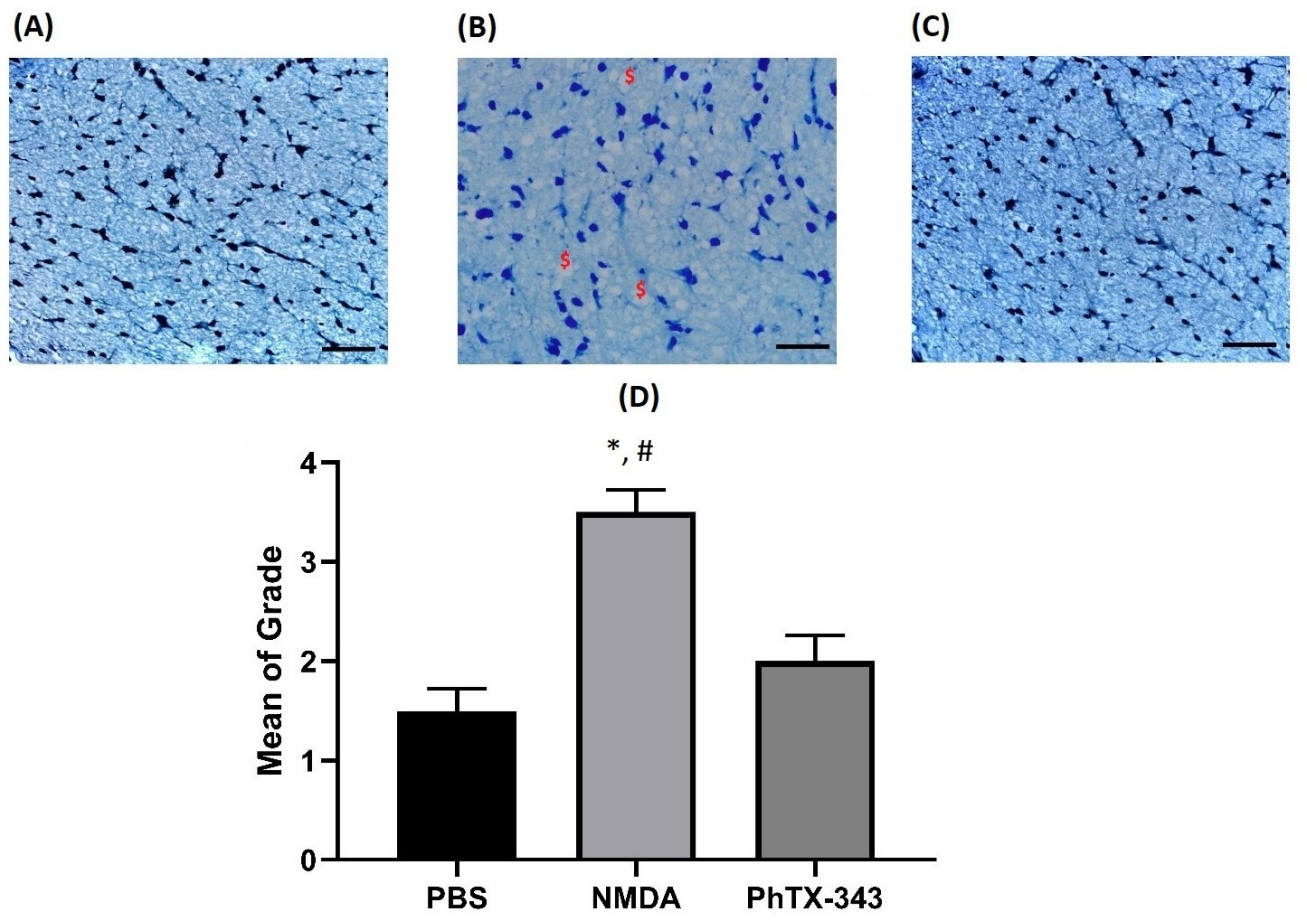
Photomicrographs of optic nerve sections from each group stained with toluidine blue seven days after intravitreal injections. (A) PBS group presenting intact optic nerve, (B) NMDA group showing extensive degeneration and vacuolation of optic nerve, (C) PhTX-343 pre-treatment group followed by NMDA administration after 24 h showing similar distribution of glial cells as in PBS group. The histological analysis was quantified by grading the extent of injury for optic nerve degeneration across the three groups and presented as bar graph (D). ($) marks degenerating fibres with vacuolation. The quantitative data were analyzed using Kruskal-Wallis with Dunn’s multiple comparison test: *p<0.05 versus PBS; #p<0.05 versus PhTX-343 (Scale bar 50 μm).

The observations on toluidine blue-stained optic nerve sections (Fig 4) were in accordance with the retinal morphology. Normal optic nerve morphology was seen in Group I (control group treated with PBS) showing packed, uniform axon fibres all over the sections. NMDA-treated group showed extensive axonal degeneration all across the sections. Pre-treatment with PhTX-343 prior to NMDA-induced injury resulted in lesser degeneration of axons with only few vacuolations, and the morphology appeared similar to the control group. These observations were supported by quantitative analysis of the optic nerve grading, demonstrating that the NMDA-induced group had the most severe degenerative changes as compared to the PBS group (p<0.05). PhTX-343 pre-treatment group exhibited less degenerative changes when compared with the NMDA group (p<0.05) while showing similar morphology as the control (p>0.05).

### Effects of PhTX-343 on exploratory and locomotor activities

**Fig 5:**
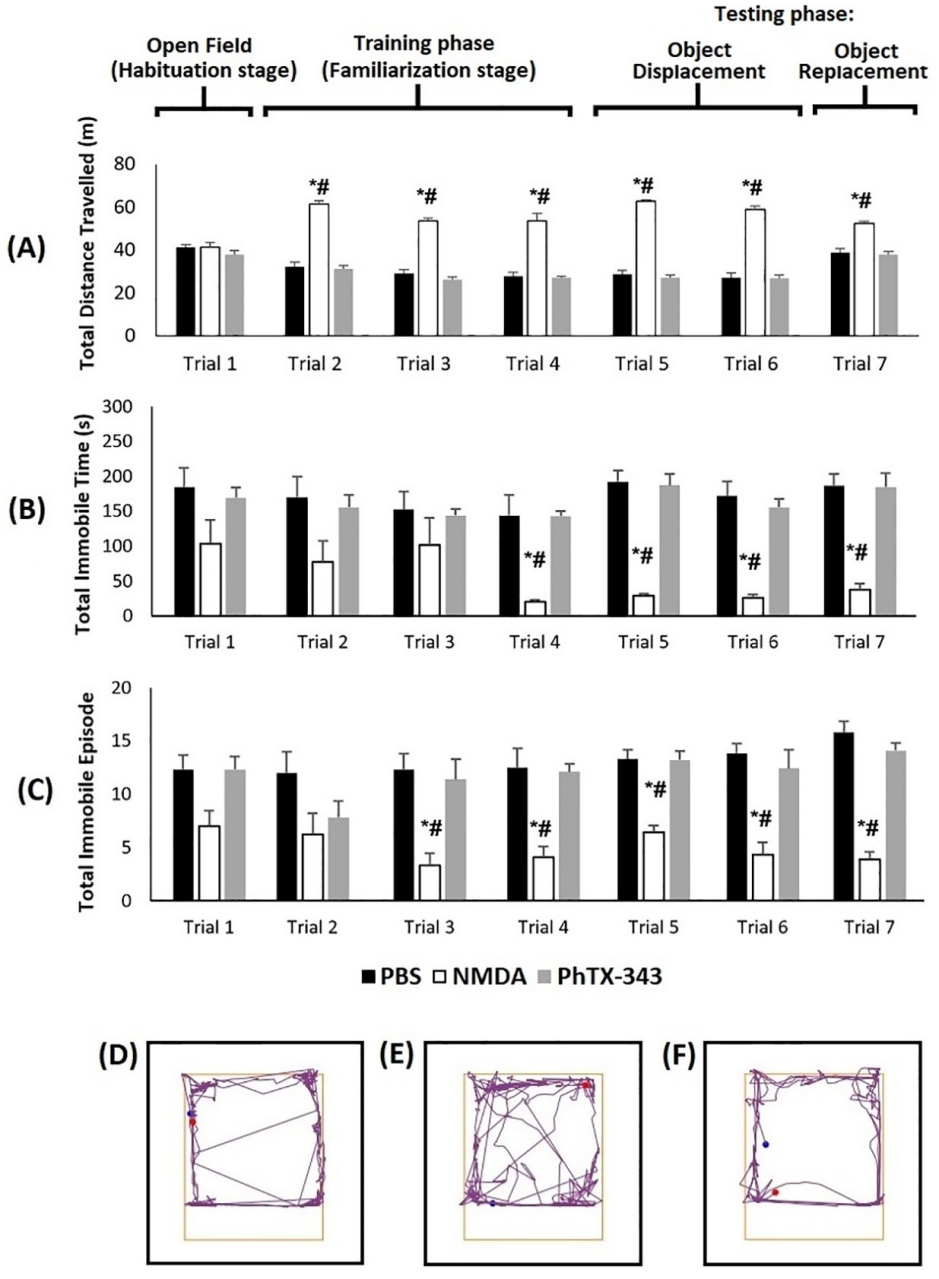
Effects of PhTX-343 on exploratory and locomotor activities using open field test in NMDA-induced rats. (A) Total distance travelled, (B) total immobile time, and (C) total immobile episodes, recorded by the rats for each group. Examples of track plots for each group are also displayed: (D) PBS, (E) NMDA, and (F) PhTX-343 groups respectively. Statistical significance was conferred using Kruskal-Wallis test with Bonferroni correction: *p<0.05 versus PBS; #p<0.05 versus PhTX-343.

As shown in Fig 5, no difference was observed in the total distance travelled, immobile time and immobile episodes from the three groups in the habituation phase (trial 1) of the open field test, suggesting that the locomotor activity of rats were comparable across all groups. However, when objects were introduced in the open field arena, the total distance travelled by rats in the PhTX-343 group were significantly lower as opposed to the NMDA group (p<0.05), but was comparable to the PBS group (p>0.05) across trials 2–7. Whereas for total immobile time and immobile episodes, the numbers recorded by the PhTX-343 group were significantly higher in contrast to the NMDA group (p<0.05) but comparable to the PBS group (p>0.05) across trials 2–7, except for trial 2 and 3 with respect to total immobile time and for trial 2 with regards to total immobile episode.

### Effects of PhTX-343 on object recognition

**Fig 6:**
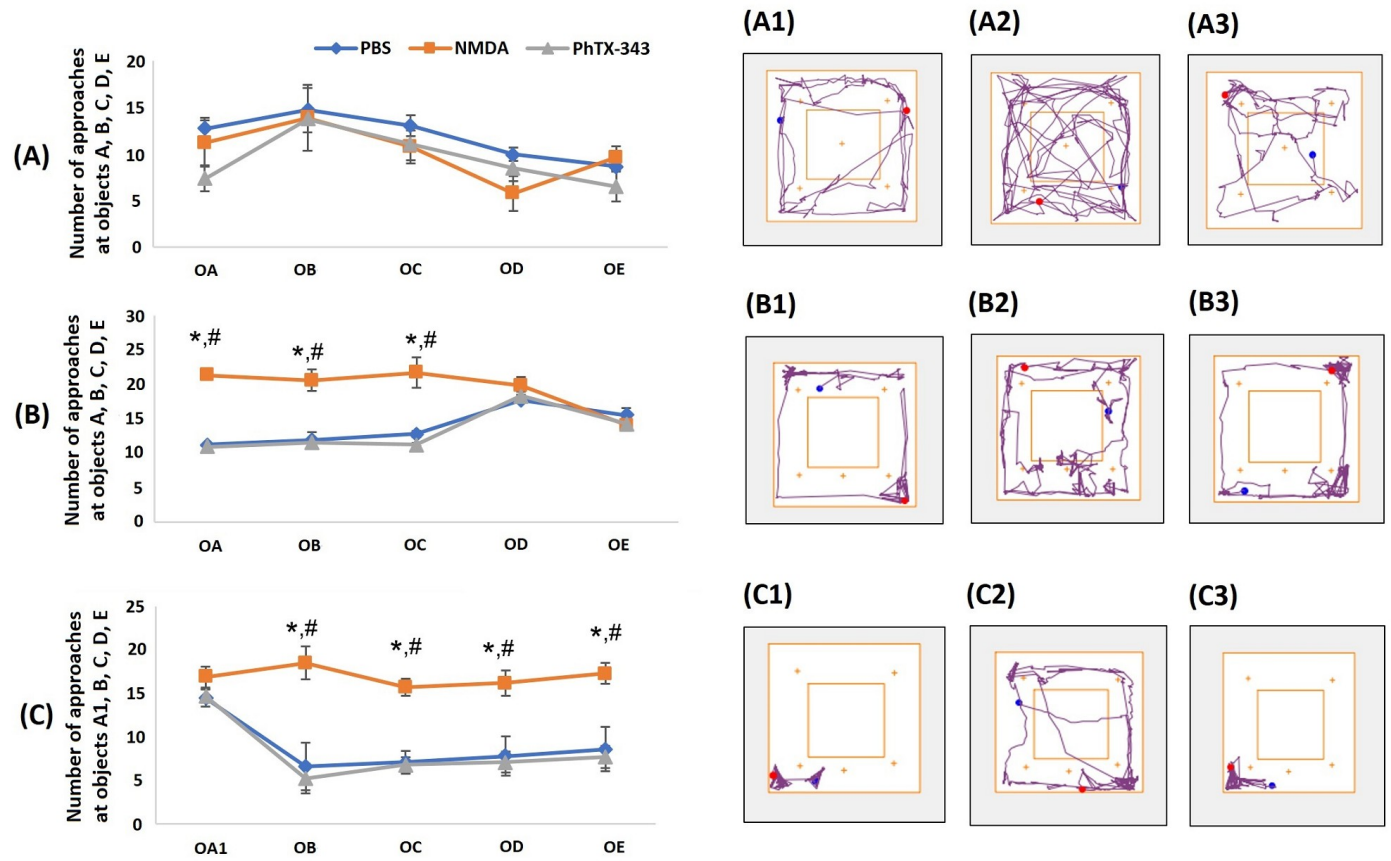
Effects of PhTX-343 on object recognition using open field arena in NMDA-induced rats. The line charts display the number of approaches by rats during: (A) familiarization phase, and examples of track plots for each group, (A1) PBS, (A2) NMDA, (A3) PhTX-343; (B) object displacement test, and examples of track plots for each group, (B1) PBS, (B2) NMDA, (B3) PhTX-343; (C) object replacement test, and examples of track plots for each group, (C1) PBS, (C2) NMDA, (C3) PhTX-343. Data were analyzed using Kruskal-Wallis test with Bonferroni correction: *p<0.05 versus PBS; #p<0.05 versus PhTX-343.

The number of time spent around novel objects were similar in all groups during the object familiarization stage of the object recognition test (Fig 6). When objects D (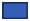) and E (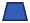) were placed in different locations during the object displacement test, rats in the NMDA-treated group exhibited a higher number of approaches at the non-displaced objects A (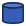), B (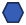), and C (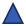) as compared to that seen for the newly repositioned objects. In contrast, both PhTX-343 and PBS groups exhibited a higher number of approaches at objects D (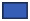) and E (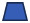) as opposed to the non-displaced objects. Comparison between groups showed that NMDA-treated rats had a higher number of approaches at the non-displaced objects A (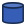), B (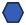), and C (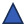) as compared to the PhTX-343 group by 2.1, 1.8 and 1.9 folds, respectively (p<0.05). This lower activity of rats observed in the PhTX-343 group was comparable to that seen in the PBS group (p>0.05).

In the object replacement stage, after object A (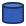) was replaced with object A1 (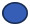), PhTX-343-treated rats showed higher exploratory activities around the new object, resulting in a significantly lower number of contacts at objects B (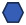), C (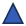), D (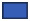), and E (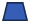) as compared to that seen for the NMDA group, corresponding to 3.6, 2.3, 2.3 and 2.3 folds, respectively (p<0.05). The number of approaches at all objects for PhTX-343 and PBS groups were comparable with no statistically significant difference.

## Discussion

Glaucoma is an ocular pathology that is characterized by the loss of RGC and optic nerve damage leading to blindness [32]. Abnormally elevated IOP is also considered a characteristic of glaucoma, however, lowering the IOP which is currently the standard practice of glaucoma management does not necessarily prevent the progression of glaucoma. Numerous reports have shown that despite IOP lowering, loss of visual field continues, and hence therapies that may provide direct neuroprotection are of considerable interest [16].

From a therapeutic standpoint, the NMDA receptors are potential targets for treatment of neurodegenerative diseases including prevention of RGC death and optic nerve damage in glaucoma [33]. As proven by early in vitro models, the influx of calcium ions into the cytoplasm as a result of NMDA receptor overstimulation may initiate the apoptosis cascade [34]. As a consequence, neuronal cell death caused by the excitotoxic conditions will eventually lead to many neurodegenerative disorders such as Alzheimer’s disease (AD), Huntington’s disease, and stroke [34, 35]. Moreover, NMDA receptors are abundantly expressed in the retina, and an in vivo model has shown that the administration of NMDA intravitreally leads to widespread apoptosis of retinal cells due to excitotoxicity (causing high cellular influx of calcium ions), which ultimately results in RGC death [32].

Currently, memantine is the only FDA-approved anti-AD drug, which targets the NMDA receptor. However, this drug failed clinical trials as a potential intervention for glaucoma [36]. The present study, reveals for the first time that pre-treatment with PhTX-343 protects against NMDA-induced retinal cell loss, optic nerve damage and visual impairment. The fact that PhTX-343 directly blocks the NMDA receptor non-competitively especially with the presence of a polyamine moiety, is presumably the reason why it exhibits neuroprotective effects [37, 38].

The present study demonstrates that pre-treatment with PhTX-343 prior to NMDA administration in rats results in reduced retinal cell loss as compared to the NMDA-treated group. In addition, the retinal morphology in the PhTX-343-treated group was comparable to that seen for the control group. In order to quantitatively assess the effect of PhTX-343 on retinal morphology, we estimated the number of retinal cells in 100 μm^2^ area as well as 100 μm length of GCL. This allowed more accurate assessment of retinal morphological changes. Cell counting only in a specified volume may not display any changes if the volume is also reduced, which appears a likely characteristic upon NMDA exposure. Conversely, cell counting during a specified length is expected to show changes despite the alteration in volume. Nevertheless, both the linear as well as volumetric density of retinal cells in NMDA-treated rat eyes were significantly lower than those observed in the control group, indicating significant retinal damage due to NMDA exposure. On the other hand, the PhTX-343 pre-treatment group showed greater density of retinal cells in the GCL as compared to that of the NMDA group, indicating its protective effects against NMDA-induced retinal injury.

In accordance with the observations made on retinal morphology, pre-treatment with PhTX-343 prior to NMDA exposure also resulted in lesser degenerative changes in the optic nerve as compared to those found for NMDA-exposed eyes, and remained comparable to control eyes. On the contrary, exposure of rat eyes to NMDA without prior PhTX-343 treatment resulted in extensive degeneration, which was characterized by swollen glial cells and clearing of nerve fibres in the entire sections. The protective effects of PhTX-343 against NMDA-induced retinal cell and optic nerve morphology observed in the present study may be attributed to its ability to counteract NMDA receptor activation in a non-competitive way, thereby reducing calcium influx and subsequent apoptosis. In fact, PhTX-343 was earlier shown to antagonize the increase in intracellular free calcium concentration in cerebellar granule cells exposed to NMDA [34]. It was also shown to inhibit NMDA-induced currents across Xenopus oocytes injected with rat brain RNA [39].

In order to assess the functional outcome of the changes in retinal and optic nerve morphology, the exploratory activities of rats were studied in the open field test. Rats that were exposed to NMDA, travelled a longer distance and were immobile for lesser time as compared to the control group, indicating their discomfort and that they require longer exploration time to familiarize with the environment. Rats that were pre-treated with PhTX-343 exhibited significantly longer periods of immobility as compared to the NMDA-treated rats indicating a higher level of comfort. Previous studies have shown that rats that recognize the visual cues get familiarized to the environment quickly, whereas those with poor vision require a longer time to adapt [40, 41]. Hence, it is hypothesized that rats receiving pre-treatment with PhTX-343 retain normal vision and recognition of visual cues, thereby enabling readily familiarization with the environment.

Subsequently, object recognition tests, involving displacement and replacement of objects, were conducted similarly to the method proposed earlier by previous study [31]. In the object displacement test, rats treated with either PhTX-343 or PBS were found to explore the newly displaced objects more than the originally positioned objects, while the NMDA-treated rats behaved totally divergent with a higher number of approaches at original objects in contrast to relocated objects. This observation evidently infers that the rats in PhTX-343 and PBS groups, which had learnt and visualized the shape and position of the objects from training during a familiarization phase, preferentially explored the newly displaced objects at novel locations rather than the original non-displaced objects [42]. This indicated that the PhTX-343 and PBS groups possess a visual ability to explore better, while rats in the NMDA group were visually impaired and only relied on their familiarized visualization of the original objects.

In the object replacement test, both PhTX-343 and PBS groups exhibited a higher number of approaches at one novel object, which was newly introduced in the arena, as compared to the other four original objects. The NMDA group, however, did not appear to show any differences between the number of approaches at all objects including the novel object. Akin to the displacement test, this result indicate that the PhTX-343 and PBS groups managed to visualize and learn the objects’ shape and their location in the arena, and hence were able to detect the novel object being introduced, whereas the NMDA group did not recognize the new object as being novel.

Our findings in both object displacement and replacement tests indicate the common trend that NMDA-treated rats explored all objects with nearly equal frequency, whereas PhTX-343 pre-treated rats explored the novel objects with higher frequency as compared to the original objects. Since the exploratory activities during the familiarization phase in open field test were almost comparable among the three groups, all the rats were expected to display similar memory of the placement of objects. However, object displacement and replacement in the study protocol made the visual cues more important than memory to recognize the familiar objects. This observation provided evidence that rats in the NMDA group had difficulties in recognizing the objects indicating visual impairments, whereas the PhTX-343 group performed similar to the control animals inferring preserved visual functions.

It is important to note that glaucoma is a multifactorial disorder. Besides NMDA receptor overactivation, other mechanisms of ganglion cell apoptosis of axonal origin such as the loss of retrograde transport of brain-derived neurotrophic factor (BDNF) from optic nerve projection sites in the brain could also contribute to the pathogenesis of glaucoma [29] and the NMDA receptor blockade alone may not be adequate enough for overall RGC protection. Nevertheless, this current study for the first time, provides evidence that PhTX-343 could be further investigated for potential use against glaucomatous RGC loss.

## Conclusion

Our data strongly suggest that PhTX-343 protects against NMDA-induced excitotoxic injuries to rat retinas. This was evidenced by prevention of NMDA-induced changes in retinal and optic nerve morphology, and by the vision-dependent behaviour of rats. Further studies are needed to explore the precise mechanisms of action of PhTX-343 against excitotoxic retinal damage.

## Supporting information

S1 File (PDF)
