# Supplementary material for: Philanthotoxin-343 attenuates retinal and optic nerve injury, and protects visual function in rats with N-methyl-D-aspartate-induced excitotoxicity
Source: PLoS One. 2020 Jul 24;15(7):e0236450. doi: 10.1371/journal.pone.0236450 (PMC7380593; doi:10.1371/journal.pone.0236450)
Supplement: S1 File — (PDF) [file pone.0236450.s001.pdf]

Histological Analysis

Group	Sample	Mean of calculated number of retinal cell nuclei/ 100 μm^2 of GCL area	Mean of calculated number of retinal cell nuclei/ 100 μm length of GCL	Mean of grading for extent of optic nerve injury
PBS	PBS(1)	0.911	11.100	2
	PBS(2)	0.929	11.271	1
	PBS(3)	0.854	10.282	1
	PBS(4)	0.899	10.754	1
	PBS(5)	1.037	12.749	2
	PBS(6)	0.919	11.970	2
NMDA	NMDA(1)	0.657	5.247	3
	NMDA(2)	0.434	3.320	3
	NMDA(3)	0.364	2.822	4
	NMDA(4)	0.625	4.461	4
	NMDA(5)	0.315	2.138	4
	NMDA(6)	0.331	2.459	3
PhTX-343	PhTX343(1)	0.852	10.011	1
	PhTX343(2)	0.754	9.624	3
	PhTX-343(3)	0.760	10.303	2
	PhTX-343(4)	0.830	10.860	2
	PhTX-343(5)	0.787	9.564	2
	PhTX-343(6)	0.743	9.777	2

Open Field Test

Group	Trials	Mean total distance travelled (m) (n=10)	Mean total immobile time (s) (n=10)	Mean total number of immobile episodes (n=10)
PBS	1	41.339	184.78	12.3
	2	32.124	170.20	12.0
	3	29.120	152.23	12.3
	4	27.795	143.81	12.5
	5	28.812	192.29	13.3
	6	27.145	171.36	13.8
	7	38.987	186.78	15.8
NMDA	1	41.424	103.59	7.0
	2	61.572	77.56	6.2
	3	53.790	101.78	3.3
	4	53.632	20.09	4.1
	5	62.740	29.09	6.4
	6	58.980	25.74	4.3
	7	52.514	37.26	3.9
PhTX-343	1	37.940	168.82	12.3
	2	31.297	155.37	7.8
	3	26.294	143.77	11.4
	4	27.044	143.15	12.1
	5	27.061	187.14	13.2
	6	26.988	155.30	12.4
	7	37.908	184.43	14.1

Object Recognition Test

Group	Objects	Familiarization Phase	Object Displacement Test	Objects	Object Replacement Test
		Average number of approaches (n=10)			Average number of approaches (n=10)
PBS	OA	12.7	11.3	OA1	14.5
	OB	14.7	11.9	OB	6.6
	OC	13.0	12.8	OC	7.1
	OD	10.0	17.8	OD	7.8
	OE	8.6	15.6	OE	8.6
NMDA	OA	11.2	21.5	OA1	16.9
	OB	13.9	20.6	OB	18.5
	OC	10.8	21.7	OC	15.7
	OD	5.7	19.8	OD	16.2
	OE	9.6	14.5	OE	17.3
PhTX-343	OA	7.3	10.9	OA1	14.7
	OB	13.7	11.6	OB	5.2
	OC	11.0	11.3	OC	6.8
	OD	8.4	18.4	OD	7.1
	OE	6.5	14.3	OE	7.7
